# Validation of blood-based detection of breast cancer highlights importance for cross-population validation

**DOI:** 10.1038/s41467-025-57265-z

**Published:** 2025-03-05

**Authors:** Bente Theeuwes, Srikant Ambatipudi, Zdenko Herceg, Chiara Maria Herzog, Martin Widschwendter

**Affiliations:** 1 European Translational Oncology Prevention and Screening Institute, Universität Innsbruck, Innsbruck, Austria; 2 Institute for Biomedical Aging Research, Universität Innsbruck, Innsbruck, Austria; 3 International Agency for Research on Cancer (IARC), Lyon, France; 4 AMCHSS, Sree Chitra Tirunal Institute for Medical Sciences and Technology, Trivandrum, India; 5 Department of Twin Research and Genetic Epidemiology, School of Life Course and Population Sciences, Faculty of Life Sciences and Medicine, King’s College London, London, UK; 6 Department of Women’s Cancer, UCL EGA Institute for Women’s Health, University College London, London, UK; 7 Department of Women’s and Children’s Health, Karolinska Institutet and Karolinska University Hospital, Stockholm, Sweden

**Keywords:** Breast cancer, Predictive markers

**arising from** T. Wang et al. *Nature Communications* 10.1038/s41467-023-40389-5 (2023)

Wang et al.^1^ recently described a new PCR-based test for breast cancer (BC) detection based on DNA methylation (DNAme) in peripheral blood-derived monocytes (PBMCs), reporting a strikingly high area under the curve (AUC) of 0.94 when combining four DNAme sites in a multiplex-based methylation-specific quantitative PCR assay. Individual loci within their multiplex assay achieved an AUC of at least 0.69 in their discovery set derived from Illumina Methylation array data. In independent whole blood methylation datasets, we observe a limited ability to distinguish BC cases from controls using these loci (*n* = 208 controls, *n* = 102 cases; maximum AUCs of 0.59 for individual, or 0.60 for combined loci, respectively), underscoring the importance of cross-population validation of diagnostic biomarkers prior to clinical implementation, although a predictor for an Asian population, even if it does not (or only weakly) validate in a European population, is still important. Notably, we show that individuals with systemic sclerosis and rheumatoid arthritis (RA) show similar changes in selected sites, suggesting that the observed signal may be at least in part associated with inflammation.

Novel diagnostic strategies for the identification of breast cancer (BC) based on molecular biomarkers may help to complement or replace existing screening modalities such as mammography that suffer from certain limitations including low sensitivity, in particular for aggressive cancers (i.e., reflected in the occurrence of interval cancers^2^), and overdiagnosis. DNAme has previously been put forward as a promising candidate for BC diagnosis, utilising either cell-free DNA^3–5^, indicative of tumour material, or anatomically distant surrogate samples such as cervical samples^6^, possibly indicative of a cancer field defect and future risk. In a recent study, Wang et al.^1^ introduced a new methylation-specific quantitative PCR assay for BC detection. The assay is based on the detection of methylation levels at four sites in peripheral blood mononuclear cells (PBMCs). Despite its simplicity, Wang et al.’s PCR-based multiplex assay reported a promising AUC of 0.94 for the distinction of early-stage BC patients and healthy controls, and exhibited sensitivities and specificities of 93.2% and 90.4% at selected thresholds, respectively. Simple and highly accurate assays have the potential to improve clinical screening and should therefore be prioritised for further evaluation and development given they prove reliable and can be validated across multiple populations. Here, we evaluate the newly identified sites in independent whole blood methylation datasets and report limited cross-population portability, which may result in diminished clinical utility.

Wang et al.^1^ identified a total of 8 candidate loci distinguishing between BC and controls, either via L1 regularisation (Lasso) or via filtering (absolute methylation difference (|Δβ| ≥ 0.08, unadjusted *p* < 0.0001). Despite their modest discovery set of 50 BC patients and 30 normal controls, in particular given the high dimensionality of the data (>800,000 sites assayed per individual), their sites largely validated across multiple sets in their study, and four were utilised in their final multiplex assay. All participant samples were derived from the same overarching cohort of participants, recruited across 10 hospitals in China. The overall AUC in the Wang methylation array discovery set, when taking the sum over the four loci selected in the final multiplex assay, was 0.76 in the methylation array (95% CI: 0.66–0.88), with individual loci ranging from 0.69 to 0.75. In the FORECEE dataset, an independent whole blood methylation dataset from 102 BC cases and 208 cancer-free healthy controls, collected across 5 hospitals and countries in Europe, 5 of the 8 loci exhibited small but significant differences (Fig. 1a, b). However, in this independent set, the overall AUC was only 0.6 (95% CI: 0.53–0.67), with values at individual CpG loci exhibiting AUCs ranging from 0.58 to 0.60 (Fig. 1c). Wang et al.’s discovery set predominantly consisted of low-stage cancers (stage 0/I BC constituted 42%). Consequently, the diagnostic value of the selected loci may be influenced by stage, and could possibly be more pronounced in early BC cases compared to late-stage cases. The FORECEE dataset was composed of ~47% low-stage cancers. Examining loci between cases and controls stratified by stage, no evident systematic reliance on cancer stage was observed (Supplementary Fig. 1a, b). Wang et al.’s claims of the potential of low-stage cancer detection, as well as their suggestion that their screening method could potentially identify tumours earlier than current clinical procedures, prompted us to also validate the loci in PLCO (Prostate, Lung, Colorectal and Ovarian Cancer Screening Trial)^7,8^ and IARC (International Agency for Research on Cancer)^9^ datasets, which contain whole-blood 450k methylation data from individuals who were later diagnosed with BC (Supplementary Fig. 2a, b). This analysis revealed only few significant differences that were not consistent with their relation to time to diagnosis. In absence of cancer stage information in the IARC dataset that could have further refined the analysis, we focused on samples collected up to 2 years pre-diagnosis, as substantial tumour growth would have been expected 1–2 years prior to diagnosis^10^. Surprisingly, the loci exhibited limited effectiveness in distinguishing cancer cases from controls, with the sum of loci AUC being 0.46 (95% CI: 0.34–0.59) for the IARC dataset and 0.49 (95% CI: 0.34–0.63) for the PLCO dataset (Supplementary Fig. 2c).

**Fig. 1:**
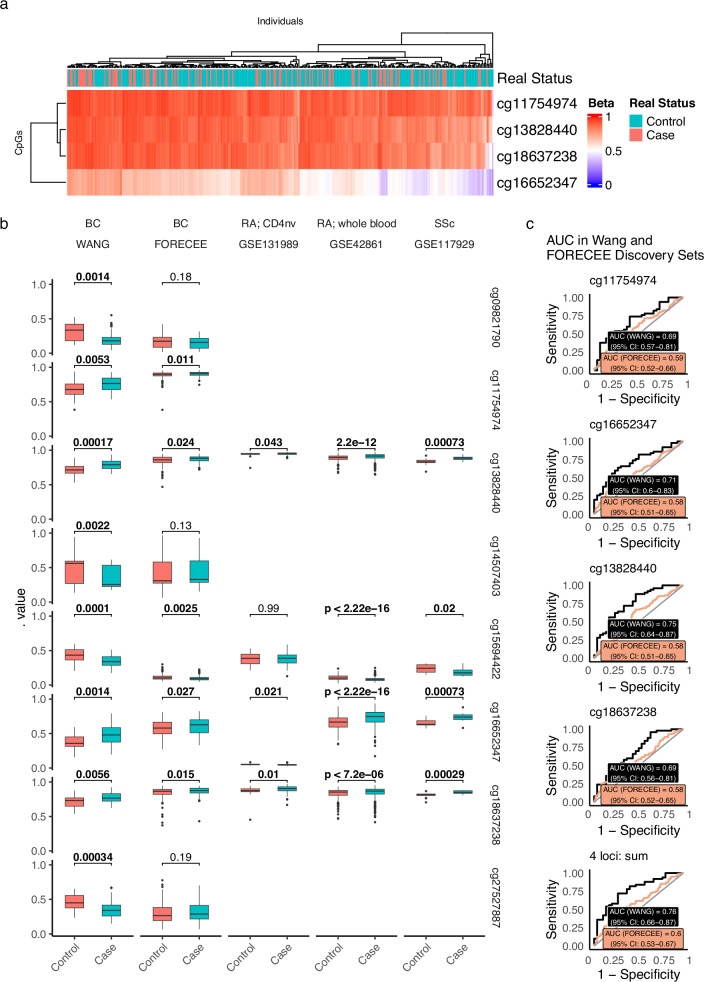
Assessing methylation of eight loci as identified by Wang et al. in the FORECEE dataset and in cases and controls of inflammatory disease. **a** Heatmap of four loci identified by Wang et al. (and implemented in their final assay) in the FORECEE dataset. **b** 8 candidate loci in breast cancer cases and controls (Wang et al. Discovery Set and FORECEE dataset), as well as in rheumatoid arthritis (RA) cases and controls (GSE131989: naive CD4 cells (CD4_nv_ cells), *n* = 123 control, *n* = 248 RA. GSE42861: whole blood, *n* = 335 control, *n* = 354 RA) and in systemic sclerosis (SSc) cases (*n* = 18) and controls (*n* = 13) (GSE117929). Differences in coverage between the 450k and EPIC arrays account for any missing data. **c** ROC curves of the four finally selected loci and their sum in the Wang Discovery Set and FORECEE datasets. Boxplots in **b** are standard Tukey representation, whereby whiskers denote minima and maxima (smallest and largest values within 1.5 times the interquartile range), the box denotes the interquartile range (25th percentile, median and 75th percentile), and dots indicate outlier values (>1.5 times the interquartile range).

We hypothesised that some of the signal in the Wang et al. study may be derived from inflammation, e.g., induced by compression mammography and/or core needle biopsy prior to the histological diagnosis of BC (e.g.^11^) rather than BC itself. As inflammation status was not available in the FORECEE data, we explored the potential association of the identified loci with inflammation by assessing methylation data from various sources: (1) a dataset from individuals with and without systemic sclerosis (SSc; whole blood)^12^, and two datasets comprising individuals with and without rheumatoid arthritis (RA; whole blood^13^ and cell subsets in peripheral blood^14^). Both systemic sclerosis and RA cases exhibit similar methylation change patterns as observed in BC cases in the dataset by Wang et al., suggesting that inflammation may, at least partially, contribute to the observed AUC (Fig. 1b). Notably, when examining the chosen loci within each specific cell subset in peripheral blood from GSE131989, it was found that the signature was present in 3 out of 4 loci in CD4+ naive T cells, while being entirely absent in all other cell types (CD14, CD19, CD4Mem) (Fig. 1b).

The use of whole blood as opposed to purified PBMCs may influence methylation results. Hence we investigated how the granulocyte proportion - a cell type that is depleted upon PBMC isolation - may affect methylation at these loci. Wang et al. reported a total granulocyte fraction of ~10% in the discovery dataset when applying the EpiDISH algorithm. Surprisingly, when we independently applied the (hierarchical) EpiDISH algorithm to their dataset, we observed a total mean granulocyte fraction of 15.8–17.8% for controls and BC cases in their dataset, respectively (Fig. 2a). Discrepancies may have arisen from the prior use of a batch correction tool by the authors, or by different reference matrix use, but detailed methods to exactly reproduce their analysis were not provided. While methylation levels did seem dependent on granulocyte fraction in the Wang discovery set, with larger differences at lower granulocyte proportion values, this was not replicated in our independent dataset (Fig. 2b, Supplementary Fig. 1c, d). Granulocyte proportion itself predicted case/control status in both the Wang and FORECEE datasets, albeit with low AUCs (0.55 and 0.61, respectively, Fig. 2c).

**Fig. 2:**
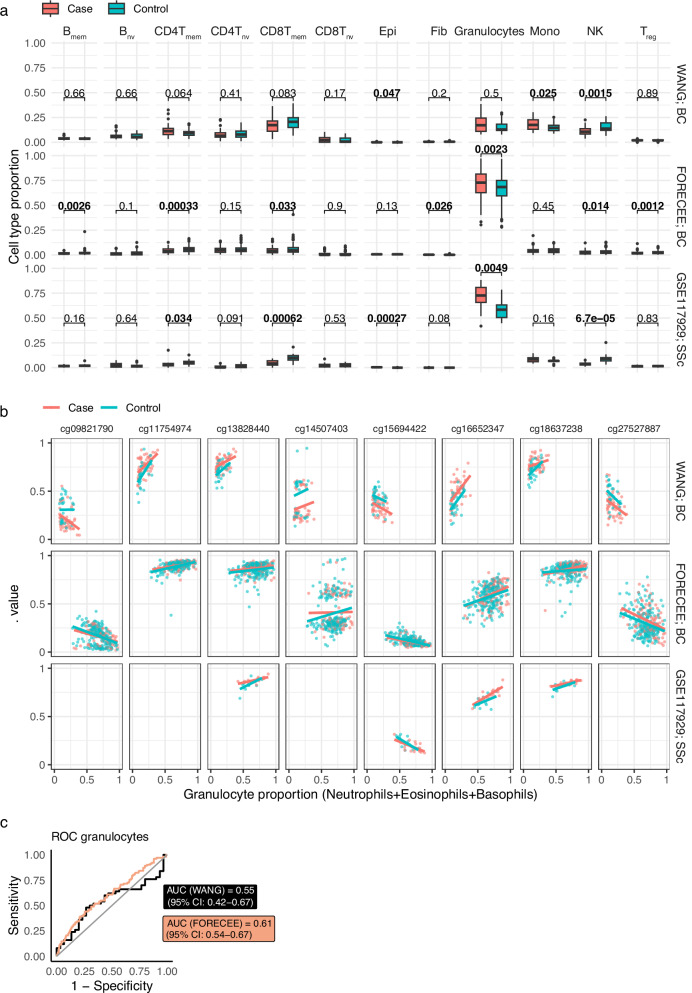
Methylation versus cell type fraction in the Wang Discovery Set and FORECEE dataset. **a** Cell type fractions inferred with EpiDISH in Breast Cancer (Wang, FORECEE; BC) and Systemic Sclerosis (GSE117929; SSc) cases and respective controls. Wang, *n* = 30 controls, *n* = 50 cases. FORECEE, *n* = 208 controls, *n* = 102 cases. GSE117929, *n* = 13 controls, 18 cases. **b** Methylation of 8 loci versus inferred granulocyte proportion. Differences in coverage between the 450k and EPIC arrays account for any missing data. **c** ROC curves of granulocyte proportion as a predictor of case/control status. Abbreviations: B_mem_ memory B cell, B_nv_ naive B cell, CD4T_mem_ memory CD4 T cell, CD4T_nv_ naive CD4 T cell, CD8T_mem_ memory CD8T cell, CD8T_nv_ naive CD8 T cell, Epi epithelial cell, Fib fibroblast, Granulocytes granulocytes (sum of neutrophils, eosinophils, and basophils), NK natural killer cell, T_reg_ regulatory T cell. Boxplots are standard Tukey representation, whereby whiskers denote minima and maxima (smallest and largest values within 1.5 times the interquartile range), the box denotes the interquartile range (25th percentile, median and 75th percentile), and dots indicate outlier values (>1.5 times the interquartile range).

We next explored differences at the newly identified loci in three breast tissue datasets (TCGA-BRCA, EPIC BC^15^ and GSE225845^16^), as Wang et al. argue that changes were exclusively driven by immune cells and not present in breast tissue. Interestingly, in the harmonised TCGA-BRCA data for BC and control tissue obtained from TCGABiolinks, only one locus (cg15694422) of the eight reported by Wang et al. was present in the data following quality control and harmonisation. This locus did not overlap with the loci described to be found in TCGA-BRCA data by Wang et al. (cg16652347, cg13828440). While we did observe a significant dependence of methylation levels on immune cell proportion and for some loci there were differences between BC tissue and normal tissue, these changes were also present in samples with very low immune cell proportions (<5%), not supporting the claim that the differential methylation at this locus is an immune-specific effect (Supplementary Fig. 3a–f; <20% in e due to low numbers). Taken together, our data indicate that while a certain dependence of methylation levels on granulocyte proportion exists in the discovery set, this was not substantiated in additional datasets and our data suggest that methylation differences are not entirely specific to immune cell proportions, albeit they are associated with inflammation (Fig. 1b).

Lastly, one of the loci discovered by Wang et al. exhibited a trimodal distribution, often indicative of single nucleotide polymorphisms or genetically determined methylation levels. Using matched genetic information in our methylation dataset, we identified that 29 genetic loci were significantly associated with methylation levels at this site at significance threshold *p* = 1e-5, possibly indicating an underlying genetic component (Supplementary Fig. 4a, b).

Our findings indicate limited cross-population portability of the sites identified by Wang et al. While some of the sites showed significant differences in the FORECEE dataset, their magnitude was much smaller. The sites exhibited no ability to distinguish BC cases from controls in samples predating cancer diagnosis (PLCO, IARC), despite the likelihood of early-stage cancers being already present in this population. Our findings imply that these markers may not offer earlier detection of BC compared to current clinical methods, as proposed by Wang et al. Additionally, their poor external performance might stem from a lack of cross-population validation. These results, and the limited portability of identified sites, could driven by one or multiple of the following: (1) limited discovery set size, identifying sites significantly different in this population by chance or underlying genetics (e.g. methylation quantitative trait loci); (2) different underlying population characteristics (e.g. genetics, ethnicity, clinicopathological features, etc.); (3) cancer signal not triggered by the presence of cancer but the inflammatory reaction occurring as a consequence of BC diagnosis (i.e., compression mammography and/or core needle biopsy or undetected conditions leading to systemic inflammation). Overall, we emphasise the need for cross-population validation and robust assessment of biomarkers across multiple clinically relevant populations (ideally based on samples from cohorts collected prior to any diagnostic manipulations), including benign conditions, prior to the development of clinical assays. Nonetheless, several factors must be considered when interpreting our additional findings: (1) the use of whole blood rather than PBMCs, although we do assess the impact of granulocytes on methylation at each site using a well-established DNAm data cellular deconvolution approach (EpiDISH); (2) we only replicated DNAm array findings and did not evaluate the PCR-based test.

## Methods

### Datasets

This research leveraged previously existing data collected in compliance with relevant ethical regulations, as described below.

DNA methylation array data (*n* = 310) and SNP data (*n* = 294, a subset of the 310) from whole blood samples derived from the FORECEE study (deposited on the European Genome-Phenome Archive [EGA] under accession number EGAS00001005055) have been described previously^17^. The multicentre FORECEE study received ethical approval from UK Health Research Authority (REC 14/LO/1633) and all contributing centres, including the NRES Committee London (UK), Ethics Committee of the General University Hospital, Prague (Czech Republic), Comitato Etico degli IRCSS Instituto Europeo di Oncologia e Centro Cardiologico Monzino (Itality), Regionale Komiteer for Medisinsk og Helsefaglig Forskningsetikk (Norway), and Ethikkommission bei der LMU München (Germany). All participants were aged >18 years and provided written informed consent. Each prospective study volunteer was given a Participant Information Sheet, as well as a Consent Form and the rationale for the study was explained. Blood DNA methylation from whole blood samples of women with BC and cancer-free controls aged 24-84 (mean age 53) were collected and processed on the Illumina MethylationEPIC array^17^, while genotypic was conducted on the Illumina 650k Infinium Global Screening Array.

Illumina MethylationEPIC array data from breast tissue samples (*n* = 56; deposited on EGA under EGAS00001005070) have been described previously^6,15,17^. In brief, this set consisted of breast tissue from premenopausal women aged 19–54 years, including normal breast tissue from 14 women who underwent cosmetic breast operations, normal breast tissue from women who underwent prophylactic mastectomies due to a *BRCA1* (*n* = 9) or a *BRCA2* (*n* = 5) mutation, and 28 samples from women who underwent surgery for triple-negative BC (for each participant, tumour tissue and tissue adjacent to the cancer was collected). All samples were collected fresh from theatre and samples processed within 1 hr of surgical excision. Fresh samples were frozen rapidly in Liquid Nitrogen and stored at −80 °C. Ethical approval was obtained from the NRES Committee East of England (reference number 15/EE/0192). All patients provided written informed consent. Samples from triple negative BCs were used as cancer cases (*n* = 14) whereas samples from normal breast tissue or normal breast tissue adjacent to a cancer were considered as controls (*n* = 42).

Harmonised and preprocessed TCGA methylation data from the TCGA-BRCA project were obtained using the R package TCGAbiolinks. A total of 889 samples were available (*n* = 792 cancer samples, *n* = 97 matched control breast tissue samples).

Data from PLCO and IARC datasets have been described previously^7–9^. PLCO data are derived from the Prostate, Lung, Colorectal and Ovarian cancer screening study. We included data from 387 incident BC cases and 359 cancer-free controls aged 55–71. The IARC dataset consisted of 902 samples from women aged 26–72, including 423 incident BC cases and 479 controls.

Data from the discovery set by Wang et al. (*n* = 80), data from systemic sclerosis cases and data from the third breast DNA methylation dataset (EPIC array) were obtained from the Gene Expression Omnibus (GEO), under accessions GSE237036, GSE117929 and GSE225845, respectively.

All data were preprocessed using our standard pipeline, eutopsQC (https://github.com/chiaraherzog/eutopsQC).

### Analysis

Statistical analysis was carried out in R version 4.2.3. Data from GEO and TCGA were accessed using GEOquery version 2.68.0 and TCGAbiolinks 2.28.3, respectively. ggplot2 version 3.4.3, ComplexHeatmap version 2.14.0 and pROC version 1.18.4 were used for data visualisations. EpiDISH version 2.14.1 was used to infer cell type proportions from processed beta matrices for the FORECEE and Wang. Specifically, the hierarchical *hepidish* function was applied, utilising centEpiFibIC.m as the primary reference matrix and cent12CT.m as the secondary reference matrix. Granulocyte proportions were computed as the cumulative proportion of basophils, neutrophils and eosinophils as inferred by hepidish. For the TCGA dataset, consisting of breast tissue, the centEpiFibFatIC.m matrix was used as reference to account for adipocytes present in breast.

SNP genotyping of a subset of blood samples in the FORECEE dataset was previously described^15^. Briefly, samples were subjected to the Illumina 650k Infinium Global Screening Array and genotypes were called using GenomeStudio followed by extensive quality control. Genetic variants associated with methylation at the putative quantitative trait locus *cg14507403* were identified using linear models implemented in MatrixEQTL^18^ version 2.3 with a significance threshold of *p* = 1e-5.

### Reporting summary

Further information on research design is available in the Nature Portfolio Reporting Summary linked to this article.

## Supplementary information

Supplementary Information

Reporting Summary

## Data Availability

Data used in these analyses are available from the following sources: Blood DNA methylation and SNP data from cancer cases and cancer-free controls in the FORECEE study is available in the European Genome-Phenome Archive (EGA) under the accession code EGAS00001005055. Blood DNA methylation data from the Wang Discovery dataset is available on NCBI Gene Expression Omnibus (GEO) under the accession GSE237036. Blood DNA methylation from rheumatoid arthritis and systemic sclerosis cases is available on NCBI GEO under the accession codes GSE131989, GSE42861 and GSE117929. Illumina MethylationEPIC breast methylation data is available in the EGA under the accession EGAS00001005070 and NCBI GEO under the accession GSE225845, while Illumina Methylation450K array data was accessed from the The Cancer Genome Atlas (TCGA) under the project accession TCGA-BRCA. Availability of DNA methylation data from the PLCO and IARC studies is described in previous publications. In brief, data are protected and not available due to data privacy laws. Access requests should be directed to respective cohort owners in writing.

## Code availability

Code to reproduce analyses presented in this study are available under https://github.com/eutops/MattersArising_BloodBC^19^.
