# Supplementary material for: Validation of blood-based detection of breast cancer highlights importance for cross-population validation
Source: Nat Commun. 2025 Mar 5;16:2164. doi: 10.1038/s41467-025-57265-z (PMC11882885; doi:10.1038/s41467-025-57265-z)
Supplement: Supplementary file 1 — Supplementary Information [file 41467_2025_57265_MOESM1_ESM.pdf]

Supplementary Information for

Validation of blood-based detection of breast cancer highlights importance for cross-population validation

Bente Theeuwes, Srikant Ambatipudi, Zdenko Herceg, Chiara Herzog and Martin Widschwendter

Supplementary Figure 1 ……………………………………….2

Supplementary Figure 2 ..…………………………………….4

Supplementary Figure 3 ………...…………………………….6

Supplementary Figure 4 ……………………………………….8

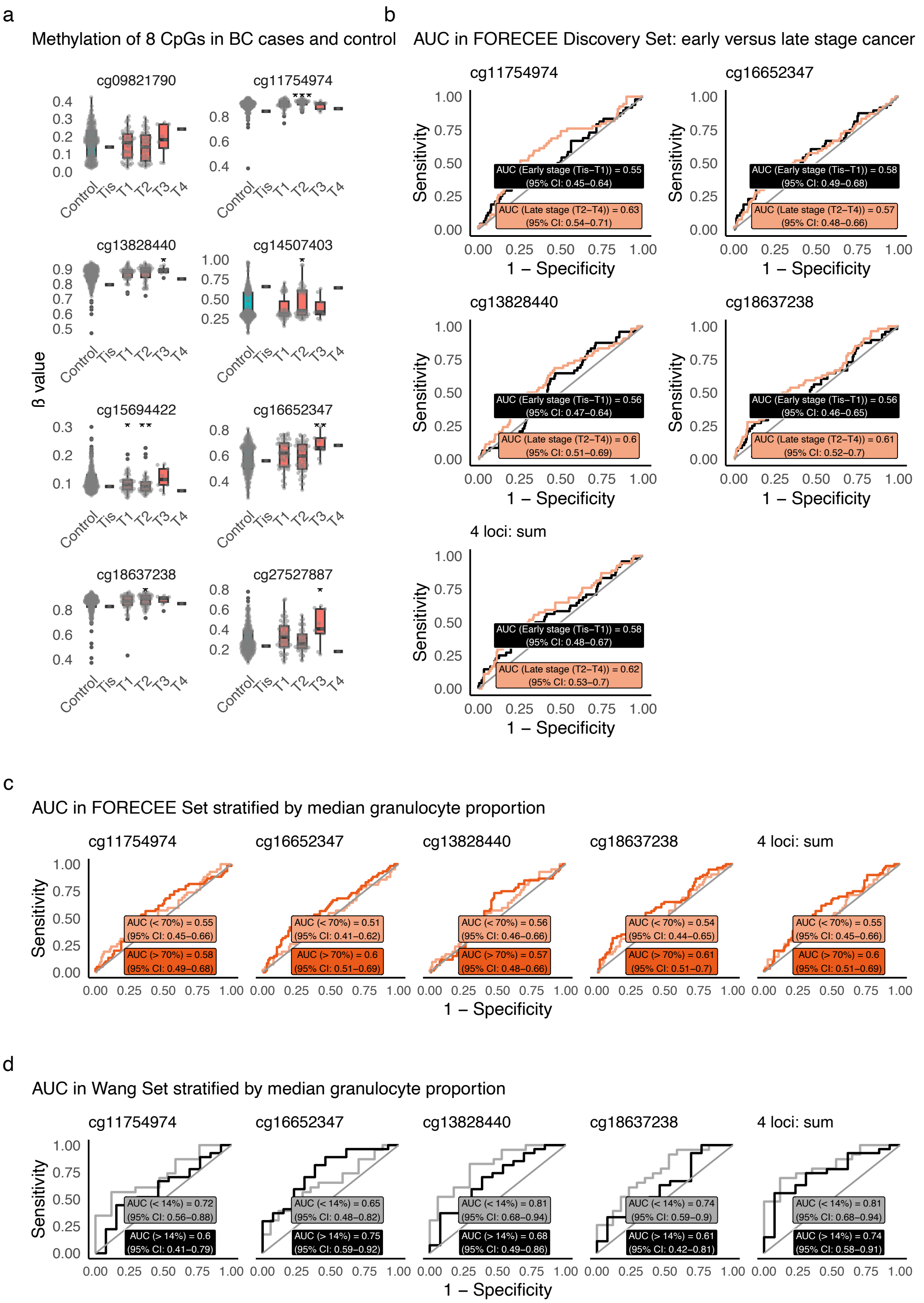

Supplementary figure 1. Assessing methylation of eight loci as identified by Wang et al. within the FORECEE dataset, stratified by cancer stage. a 8 candidate loci in breast cancer cases and controls in the FORECEE set, stratified by cancer stage. Individual datapoints are overlaid. Sample numbers were as follows: Controls, n=208; Tis, n = 1; T1, n=47; T2, n=44; T3, n=9; T4, n=1. **b** ROC curves of the four finally selected loci and their sum in the FORECEE dataset, stratified by early stage (Tis-T1, n=48) and late stage (T2-T4, n=54) cancers versus controls (n=208). **c** ROC curves of the four finally selected loci and their sum in the FORECEE dataset, stratified by median granulocyte proportion (70%) in the same data set (low granulocyte proportion:

n=113 controls, n=42 cases; high granulocyte proportion: n=95 controls, n=60 cases). **d** ROC curves of the four finally selected loci and their sum in the Wang dataset, stratified by median granulocyte proportion (14%) in the same data set. (low granulocyte proportion: n=17 controls, n=23 cases; high granulocyte proportion: n=13 controls, n=27 cases).

Boxplots are standard Tukey representation, whereby whiskers denote minima and maxima (smallest and largest values within 1.5 times the interquartile range), the box denotes the interquartile range (25th percentile, median, and 75th percentile), and dots indicate outlier values (>1.5 times the interquartile range). P values are derived from two-sided Wilcoxon tests compared to Control values, with *, **, ***, and **** representing p<0.05, <0.01, <0.001, <0.0001, respectively. No adjustment for multiple testing was applied.

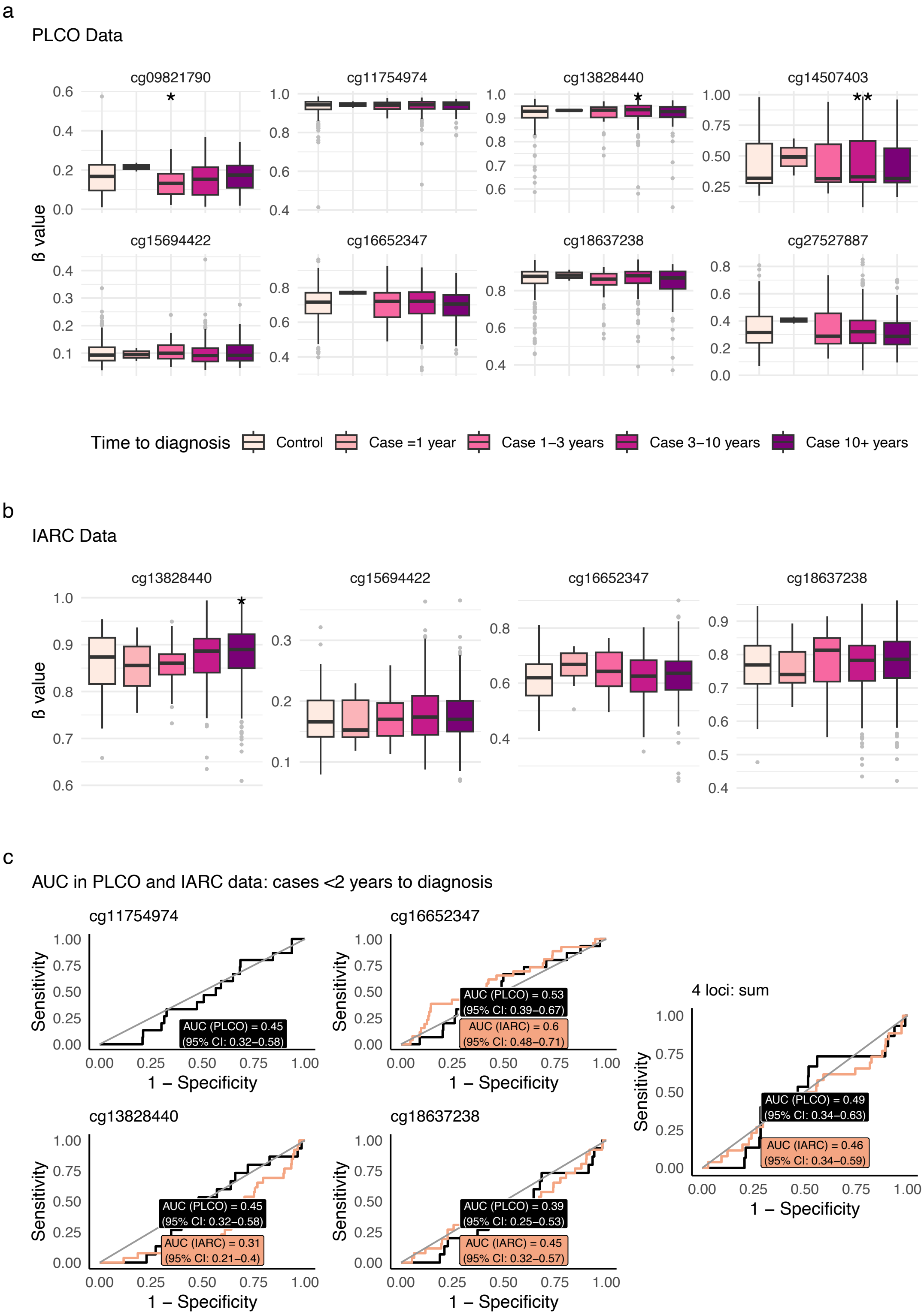

Supplementary figure 2. Assessing methylation of eight loci as identified by Wang et al. within the datasets of controls and future breast cancer cases in dataset derived from the International Agency of Research on Cancer (IARC) and Prostate, Lung, Colorectal, and Ovarian Cancer Screening (PLCO). a Methylation versus time to diagnosis in the PLCO dataset (*n=359* control, *n=387* BC case). **b** Methylation versus time to diagnosis in the IARC dataset (*n=479* control, *n=423* BC case). **c** ROC curves of the four loci and their sum in the PLCO dataset and the IARC dataset for control and cases with less than 2 years to the time of diagnosis. For the PLCO dataset only late-stage cancers were taken into account (>T1); due to the

unavailability of such data, all stages were included for AUC calculation in the IARC dataset (IARC: *n=479* control, *n=26* BC case; PLCO: *n=359* control, *n=15* BC case).

Boxplots are standard Tukey representation, whereby whiskers denote minima and maxima (smallest and largest values within 1.5 times the interquartile range), the box denotes the interquartile range (25th percentile, median, and 75th percentile), and dots indicate outlier values (>1.5 times the interquartile range). P values are derived from two-sided Wilcoxon tests compared to Control values, with *, **, ***, and **** representing $p<0.05$, <0.01, <0.001, <0.0001, respectively. No adjustment for multiple testing was applied.

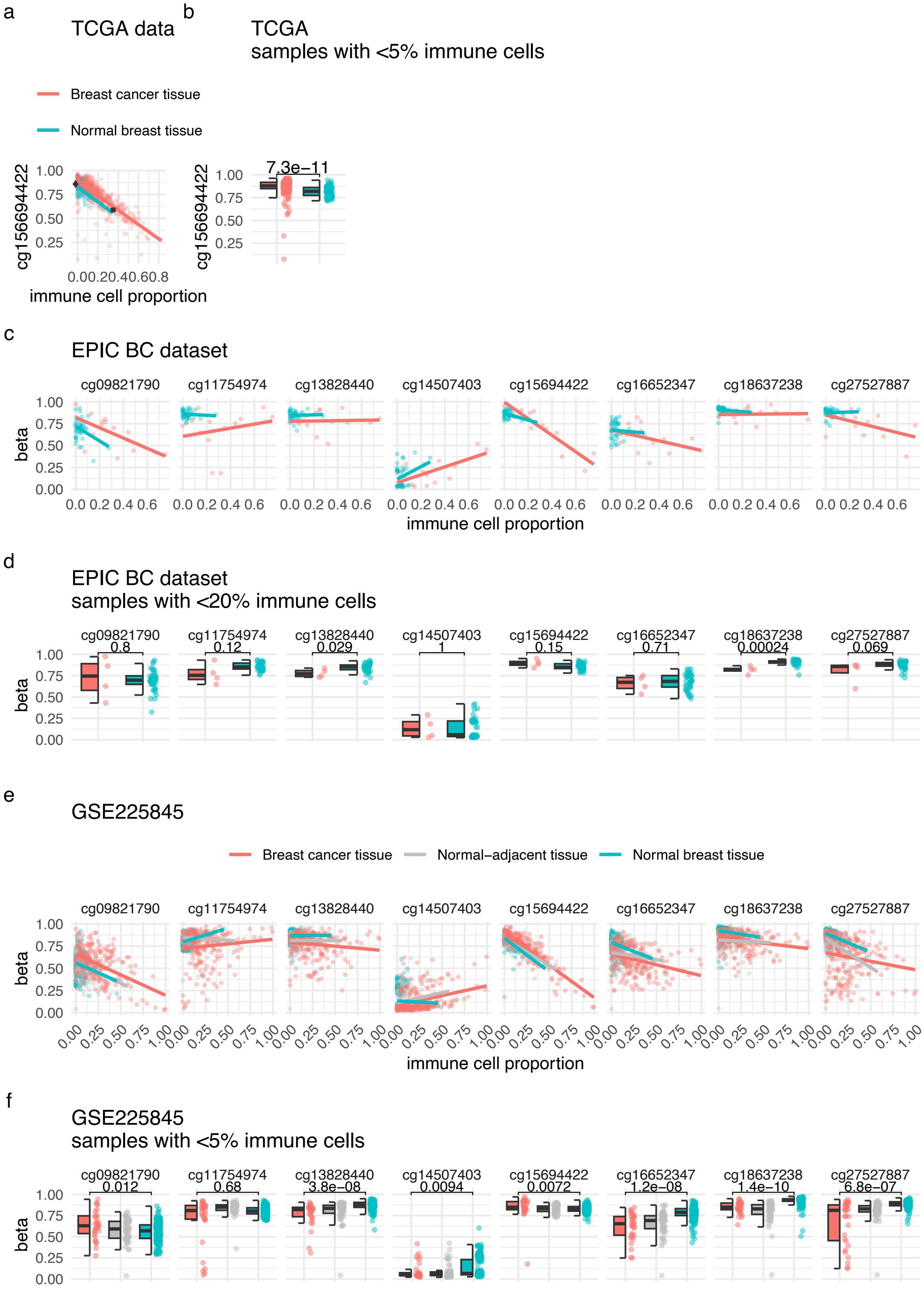

Supplementary figure 3. Assessing methylation of eight loci as identified by Wang et al. in breast tissue. a Methylation versus immune cell fraction for cg15694422 in the TCGA dataset, comparing breast cancer tissue (n=792) with normal tissue (n=97). **b** Boxplot showing methylation differences for cg15694422

between breast cancer and normal tissues in the TCGA dataset, focusing on samples with immune cell fractions <5% (n=65 controls, n=181 cases). **c** Methylation versus immune cell fraction across eight loci identified by Wang et al., analysed in an additional dataset of normal adjacent and triple-negative breast cancer tissues ("EPIC BC dataset"; n=14 cases, n=42 controls). **d** Methylation versus immune cell fraction across the eight loci in the same additional dataset, focusing on samples with immune cell fractions <20% (n=4 cases, n=41 controls). **e** Comparison of methylation versus immune cell fraction for eight loci identified by Wang et al. in the GSE225845 dataset, examining breast cancer (n=224), normal-adjacent (n=140), and normal breast tissues (n=231). **f** Methylation versus immune cell fraction for the same eight loci in GSE225845, restricted to samples with immune cell fractions <5%, comparing breast cancer (n=30), normal-adjacent (n=55), and normal breast tissues (n=162).

Boxplots are standard Tukey representation, whereby whiskers denote minima and maxima (smallest and largest values within 1.5 times the interquartile range), the box denotes the interquartile range (25th percentile, median, and 75th percentile), and dots indicate outlier values (>1.5 times the interquartile range). P values are derived from two-sided Wilcoxon tests compared to Breast cancer tissue values, with *, **, ***, and **** representing p<0.05, <0.01, <0.001, <0.0001, respectively. No adjustment for multiple testing was applied.

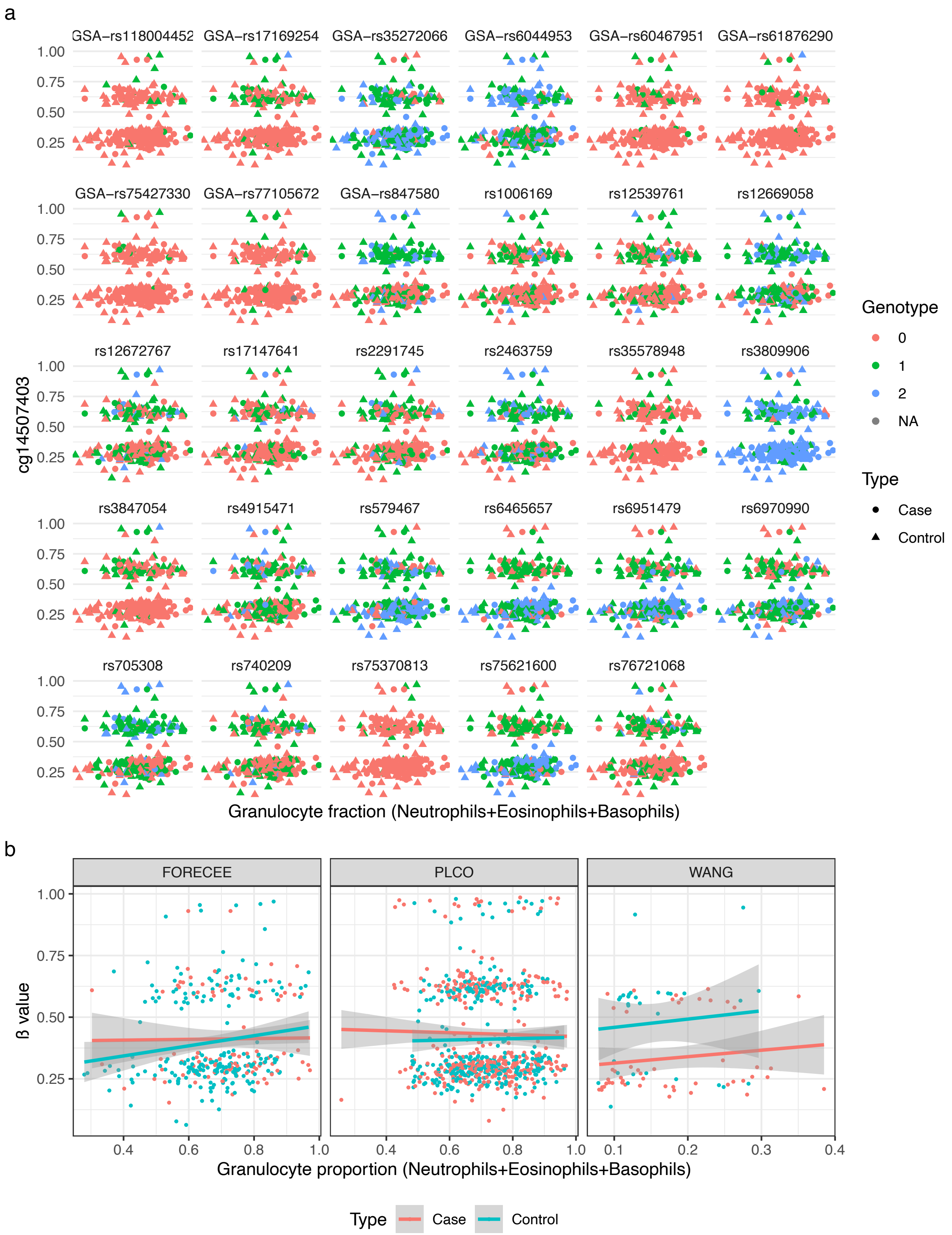

Supplementary figure 4. *cg14507403* exhibits a trimodal distribution and might be indicative of single nucleotide polymorphisms or genetically determined methylation levels. a Methylation versus immune cell fraction for *cg14507403* in the FORECEE dataset (n=294), and its association with individual single nucleotide polymorphisms (p<1e-5 in EWAS). **b** Methylation of *cg14507403* versus inferred granulocyte proportion in three datasets evaluated. Data are coloured by case or control status.
