# Supplementary material for: Validation of blood-based detection of breast cancer highlights importance for cross-population validation
Source: Nat Commun. 2025 Mar 5;16:2164. doi: 10.1038/s41467-025-57265-z (PMC11882885; doi:10.1038/s41467-025-57265-z)
Supplement: Supplementary file 2 — Reporting Summary [file 41467_2025_57265_MOESM2_ESM.pdf]

nature portfolio

Corresponding author(s): Martin Widschwendter and Chiara Herzog

Last updated by author(s): 12/18/2024

Reporting Summary

Nature Portfolio wishes to improve the reproducibility of the work that we publish. This form provides structure for consistency and transparency in reporting. For further information on Nature Portfolio policies, see our Editorial Policies and the Editorial Policy Checklist.

Statistics

For all statistical analyses, confirm that the following items are present in the figure legend, table legend, main text, or Methods section.

n/a	Confirmed	
☐	☒	The exact sample size (*n*) for each experimental group/condition, given as a discrete number and unit of measurement
☐	☒	A statement on whether measurements were taken from distinct samples or whether the same sample was measured repeatedly
☐	☒	The statistical test(s) used AND whether they are one- or two-sided<br>*Only common tests should be described solely by name; describe more complex techniques in the Methods section.*
☐	☒	A description of all covariates tested
☐	☒	A description of any assumptions or corrections, such as tests of normality and adjustment for multiple comparisons
☐	☒	A full description of the statistical parameters including central tendency (e.g. means) or other basic estimates (e.g. regression coefficient) AND variation (e.g. standard deviation) or associated estimates of uncertainty (e.g. confidence intervals)
☐	☒	For null hypothesis testing, the test statistic (e.g. *F*, *t*, *r*) with confidence intervals, effect sizes, degrees of freedom and *P* value noted<br>*Give P values as exact values whenever suitable.*
☒	☐	For Bayesian analysis, information on the choice of priors and Markov chain Monte Carlo settings
☒	☐	For hierarchical and complex designs, identification of the appropriate level for tests and full reporting of outcomes
☐	☒	Estimates of effect sizes (e.g. Cohen's *d*, Pearson's *r*), indicating how they were calculated

Our web collection on statistics for biologists contains articles on many of the points above.

Software and code

Policy information about availability of computer code

Data collection	Data were obtained from existing sources; no new data collection was conducted.
Data analysis	The preprocessing of the EPIC array datas was done using our standard pipeline, eutopsQC (https://github.com/chiaraherzog/eutopsQC). Statistical analysis was carried out in R version 4.2.3. Data from GEO and TCGA were accessed using GEOquery version 2.68.0 and TCGAbiolinks 2.28.3, respectively. ggplot2 version 3.4.3, ComplexHeatmap version 2.14.0, and pROC version 1.18.4 were used for data visualisations. EpiDISH version 2.14.1 was used to infer cell type proportions from processed beta matrices for the FORECEE and Wang. Specifically, the hierarchical hepidish function was applied, utilising centEpiFibIC.m as the primary reference matrix and cent12CT.m as the secondary reference matrix. Granulocyte proportions were computed as the cumulative proportion of basophils, neutrophils, and eosinophils as inferred by hepidish. For the TCGA dataset, consisting of breast tissue, the centEpiFibFatIC.m matrix was used as reference to account for adipocytes present in breast.<br><br>SNP genotyping of a subset of blood samples in the FORECEE dataset was previously described 15. Briefly, samples were subjected to the Illumina 650k Infinium Global Screening Array and genotypes were called using GenomeStudio followed by extensive quality control. Genetic variants associated with methylation at the putative quantitative trait locus cg14507403 were identified using linear models implemented in MatrixEQTL 20 version 2.3 with a significance threshold of p=1e-5.<br><br>Code to reproduce analyses presented in this study are available under https://github.com/eutops/MattersArising_BloodBC [10.5281/zenodo.14514726].

For manuscripts utilizing custom algorithms or software that are central to the research but not yet described in published literature, software must be made available to editors and reviewers. We strongly encourage code deposition in a community repository (e.g. GitHub). See the Nature Portfolio guidelines for submitting code & software for further information.

Data

Policy information about availability of data

All manuscripts must include a data availability statement. This statement should provide the following information, where applicable:

- Accession codes, unique identifiers, or web links for publicly available datasets
- A description of any restrictions on data availability
- For clinical datasets or third party data, please ensure that the statement adheres to our policy

Data used in these analyses are available from the following sources: Blood DNA methylation and SNP data from cancer cases and cancer-free controls in the FORECEE study is available in the European Genome-Phenome Archive (EGA) under the accession code EGAS00001005055 [https://ega-archive.org/studies/EGAS00001005055]. Blood DNA methylation data from the Wang Discovery dataset is available on NCBI Gene Expression Omnibus (GEO) under the accession GSE237036 [https://www.ncbi.nlm.nih.gov/geo/query/acc.cgi?acc=GSE237036]. Blood DNA methylation from rheumatoid arthritis and systemic sclerosis cases is available on NCBI GEO under the accession codes GSE131989 [https://www.ncbi.nlm.nih.gov/geo/query/acc.cgi?acc=GSE131989], GSE42861 [https://www.ncbi.nlm.nih.gov/geo/query/acc.cgi?acc=GSE42861], and GSE117929 [https://www.ncbi.nlm.nih.gov/geo/query/acc.cgi?acc=GSE117929]. Illumina MethylationEPIC breast methylation data is available in the EGA under the accession EGAS00001005070 [https://ega-archive.org/studies/EGAS00001005070] and NCBI GEO under the accession GSE225845 [https://www.ncbi.nlm.nih.gov/geo/query/acc.cgi?acc=GSE225845], while Illumina Methylation450K array data was accessed from the The Cancer Genome Atlas (TCGA) under the project accession TCGA-BRCA [https://portal.gdc.cancer.gov/projects/TCGA-BRCA]. Availability of DNA methylation data from the PLCO and IARC studies is described in previous publications. In brief, data are protected and not available due to data privacy laws. Access requests should be directed to respective cohort owners in writing.

Research involving human participants, their data, or biological material

Policy information about studies with human participants or human data. See also policy information about sex, gender (identity/presentation), and sexual orientation and race, ethnicity and racism.

Reporting on sex and gender	Due to the disease studied (i.e. breast cancer in women) only women were included in this study.
Reporting on race, ethnicity, or other socially relevant groupings	No analysis based on ethnicity, race, or other socially relevant groupings was conducted in the current study.
Population characteristics	All participants were female, and population descriptions have been extensively described in original studies of these datasets. Age ranges are described in the current manuscript. The population included women with a current or future diagnosis of breast cancer or cancer-free controls, derived from FORECEE, IARC, PLCO, and Wang datasets, as well as TCGA.
Recruitment	No recruitment was conducted in the current study.
Ethics oversight	The current study did not require additional ethical approval and used existing data only. All studies generating underlying data have received ethical approval from relevant institutions.

Note that full information on the approval of the study protocol must also be provided in the manuscript.

Field-specific reporting

Please select the one below that is the best fit for your research. If you are not sure, read the appropriate sections before making your selection.

☒ Life sciences ☐ Behavioural & social sciences ☐ Ecological, evolutionary & environmental sciences

For a reference copy of the document with all sections, see nature.com/documents/nr-reporting-summary-flat.pdf

Life sciences study design

All studies must disclose on these points even when the disclosure is negative.

Sample size	No sample size calculation was conducted for the current study. This study leveraged existing data for replication of findings by Wang et al.
Data exclusions	No data were excluded.
Replication	The current study is a replication/validation study (Matter arising). We attempt to replicate findings by Wang et al. and show that in additional, largely European cohort, findings do not replicate.
Randomization	The study did not include any intervention or experimental group, hence no randomization was required.
Blinding	No blinding was conducted as this study only conducted an analysis of existing data.

Reporting for specific materials, systems and methods

We require information from authors about some types of materials, experimental systems and methods used in many studies. Here, indicate whether each material, system or method listed is relevant to your study. If you are not sure if a list item applies to your research, read the appropriate section before selecting a response.

Materials & experimental systems

n/a	Involved in the study
☒	☐ Antibodies
☒	☐ Eukaryotic cell lines
☒	☐ Palaeontology and archaeology
☒	☐ Animals and other organisms
☒	☐ Clinical data
☒	☐ Dual use research of concern
☒	☐ Plants

Methods

n/a	Involved in the study
☒	☐ ChIP-seq
☒	☐ Flow cytometry
☒	☐ MRI-based neuroimaging

Plants

Seed stocks	*Report on the source of all seed stocks or other plant material used. If applicable, state the seed stock centre and catalogue number. If plant specimens were collected from the field, describe the collection location, date and sampling procedures.*
Novel plant genotypes	*Describe the methods by which all novel plant genotypes were produced. This includes those generated by transgenic approaches, gene editing, chemical/radiation-based mutagenesis and hybridization. For transgenic lines, describe the transformation method, the number of independent lines analyzed and the generation upon which experiments were performed. For gene-edited lines, describe the editor used, the endogenous sequence targeted for editing, the targeting guide RNA sequence (if applicable) and how the editor was applied.*
Authentication	*Describe any authentication procedures for each seed stock used or novel genotype generated. Describe any experiments used to assess the effect of a mutation and, where applicable, how potential secondary effects (e.g. second site T-DNA insertions, mosiacism, off-target gene editing) were examined.*
